# Cancer care coordination determinants of depression in head and neck cancer survivors

**DOI:** 10.1007/s00520-025-09847-2

**Published:** 2025-08-20

**Authors:** Yi-Ping Wen, Kristen R. Choi, Eden R. Brauer

**Affiliations:** 1 School of Nursing, University of California, 700 Tiverton Ave, Los Angeles, CA 90095 USA; 2 Department of Health Policy and Management, Fielding School of Public Health, University of California, Los Angeles, CA USA

**Keywords:** Head and neck neoplasms, Cancer care coordination, Depression, Supervised machine learning, Mental health, Cancer survivor

## Abstract

**Purpose:**

This study aims to explore the role of patient-reported cancer care coordination in explaining depression among head and neck (HNC) cancer survivors.

**Methods:**

In this cross-sectional study, the University of California Los Angeles Health tumor registry was used to recruit English-proficient adult HNC survivors who were at least 1-year post-diagnosis. Participants completed the Cancer Care Coordination Questionnaire for Patients (CCCQ-P) and the Patient Reported Outcomes Measurement Information Systems (PROMIS) Short Form v1.0, Depression 4a, as part of a comprehensive survey. Data were analyzed using the Generalized Boosted Regression Models.

**Results:**

A total of 347 HNC survivors participated, with the majority being male (66.6%), white (81.5%), married (70.6%), and on average 44.4 months since cancer diagnosis. The average CCCQ-P scores were 74.6 (SD = 14.6). Factors associated with depression included confusion regarding healthcare professionals’ roles, insufficient support from oncology staff, overall ratings of cancer care coordination, the absence of a caregiver, and being in mid-adulthood.

**Conclusions:**

We identified correlates of depression among HNC survivors related to the patient care experience. This finding suggests that improving care coordination may increase opportunities to improve HNC survivors’ mental health outcomes and long-term quality of life.

**Implications:**

Clear clinical pathways are essential for linking patient needs to care resources. Tools that efficiently identify patient concerns, like the NCCN distress thermometer and patient concerns inventory, and connect them to algorithms for tailored support should be considered for implementation. Patient navigators may also enhance care coordination by clarifying specific roles of the multidisciplinary team, improving communication and reducing care delays.

**Supplementary Information:**

The online version contains supplementary material available at 10.1007/s00520-025-09847-2.

## Background

Head and neck cancers (HNCs) are heterogeneous, malignant growths originating from the head and neck regions, including the oral cavity, throat (pharynx), larynx, sinonasal, and salivary glands [1]. In 2024, an estimated 58,450 new cases and 12,230 deaths in HNC in the United States (US) accounted for 3% of all new cancer cases [2, 3]. The 5-year relative survival varies widely by cancer stage, from 87.5% for localized to 37.8% for distant disease [3]. Contemporary HNC treatment often involves a combination of multimodal regimens, including surgery, radiation, and chemoimmunotherapy [4], which can greatly affect HNC survivors’ facial appearance, sensory nerves, taste buds, and functional status with respect to speaking and swallowing, as well as their mental health.

Patients with HNC face an increased risk of depression, anxiety, and suicidality compared to both the general population and other cancer patient populations [5–7]. In a population-based study of 14,054 HNC survivors, 64.2% suffered from depressive symptoms [8]. Findings from this study indicated that the onset of depression peaked during the post-treatment period [9], and 30–40% reported persistent depressive mood 12 months post-diagnosis [10]. In addition to psychological challenges, depression has been linked to low treatment adherence [11], cancer progression [12], and worse overall survival in HNC survivors [9, 13]. Given high risk for post-treatment depression, it is important to understand how patient experiences of care affect depression symptoms in this population.

Achieving optimal mental health outcomes necessitates highly coordinated care across a multidisciplinary team for accurate diagnosis, treatment planning, and post-treatment recovery, support and rehabilitation [1]. Care coordination refers to “the deliberate organization of patient care activities between two or more participants (including the patient) involved in a patient’s care to facilitate the appropriate delivery of health care services” [14]. In the context of head and neck cancer, which often requires multimodal therapy and extensive post-treatment rehabilitation, care coordination is becoming more complicated. Patients and their families frequently face the challenge of managing numerous healthcare interactions with disparate clinical teams and settings over a prolonged period of time [15]. Patients receiving oncological treatment in community settings often have difficulty identifying and coordinating their care with a multidisciplinary healthcare team [16]. The disconnect between cancer and mental health services, particularly for patients undergoing treatment in community settings, complicates care coordination. Therefore, better understanding of patients’ experiences of cancer care coordination (CCC) and its role in mental health outcomes is warranted.

There is already some evidence that improving CCC can enhance cancer survivors’ well-being. For example, one study found that community practices that participated in the oncology care model (OCM) and received incentivized payments for improving CCC reported fewer cancer office visits and reduced cancer care costs [17]. Implementation of nurse-delivered cancer navigation has also shown reductions in time from cancer diagnosis to oncology consultation and a 6-month reduction in avoidable primary care provider visits after oncology referral [18]. Conversely, the absence of a patient navigator was associated with poorer perceptions of CCC among both patients and their caregivers [19]. A small number of studies have linked CCC to the incidence of depression among patients with cancer [20, 21]. In the specific context of HNC, the number of unmet cancer emotional and information needs have been positively correlated with poor outcomes, including depression [22]. Collaborative care interventions, such as those combining symptom management education with coordination, have been shown to significantly reduce depressive symptoms among survivors with pre-existing depression, including those with advanced-stage cancer [23]. However, existing studies have been limited by small sample sizes, were not conducted in the US [20], focused on CCC experiences in palliative care settings [23], and did not examine patients’ experience with CCC comprehensively [22]. There is a need to better understand the specific CCC factors associated with depression risk so that these factors can be targeted with interventions.

Thus, this study aims to examine HNC survivors’ CCC experiences and their relationship to depression. We hypothesized that specific CCC experiences are critical in influencing the mental well-being of HNC survivors. Identifying these specific CCC factors associated with depression has considerable potential to tailor interventions aimed at improving their mental health outcomes.

## Methods

### Design and conceptual framework

This was a cross-sectional study of HNC survivors who were at least 1 year post-diagnosis. We focused on the post-diagnosis period because this is when depression risk tends to increase. The framework for this study was guided by Gelberg and Andersen’s behavioral model for vulnerable populations [24]. In this model, an individual’s health outcome is driven by predisposing sociodemographic characteristics, enabling resources, and their perceived or evaluated care needs (Supplemental Material 1). When applied to this study, the model guided the examination of sociodemographic and clinical factors, such as age, gender, marital status, annual household income, insurance status, and cancer stage, as well as CCC experiences that might explain depression among HNC survivors.

### Participants and settings

Potentially eligible participants were identified through the UCLA health tumor registry. Adults who received a diagnosis of HNC, including cancers of the pharynx, larynx, tonsils, salivary glands, and oral cavity at least 1 year before the study and could read and answer questions in English were eligible. Eligible participants were contacted via postal or electronic mail and invited to participate.

### Data collection

Recruitment and data collection took place from November 2019 to April 2020. Participants were asked to complete a comprehensive set of questionnaires, which included the Cancer Care Coordination Questionnaire for Patients (CCCQ-P), sociodemographic, and clinical characteristics. Patients were sent a unique survey link via the Research Electronic Data Capture (REDCap), and their responses were entered directly into the database. Hardcopy surveys and return envelopes with prepaid postage were provided when requested. Informed consent was obtained from all participants through the submission of their completed surveys, indicating their voluntary agreement to participate in this study.

### Measures

#### Cancer care coordination questionnaire for patients

The Cancer Care Coordination Questionnaire for Patients (CCCQ-P) was developed in 2011 to provide a reliable measurement of patients’ experiences with CCC in their cancer care journey [25]. This measure has been validated in patients with lung, colorectal, gynecological, and breast cancers and those undergoing or having completed cancer therapy [25, 26], with internal consistency measured by Cronbach’s alpha of 0.88 [25]. The CCCQ-P is a 22-item questionnaire that generates a total score based on 20 items between 20 and 100 and two subscale scores, “communication” (items 1–13, possible score range 13–65) and “navigation” (items 14–20, possible score range 7–35). Each item uses a 5-point Likert response scale ranging from 1 to 5. Higher scores between questions 1–13 and 14–20 indicate higher and lower satisfaction, respectively. Items 14–20 were reverse coded for the convenience of result interpretation. In addition, two items (items 21 and 22) assess respondents’ overall ratings of CCC and care satisfaction, using a scale of 1 to 10, where higher scores represent a better perception of care. More specifically, the communication subscale incorporates several aspects of cancer care topics, such as communication on cancer signs and symptoms (item 1), cancer tests and treatment required (item 2), rationale of cancer treatment choices, treatment benefits and side effects (items 3–5), access to counseling services (item 6), emotional support from healthcare team (items 7 and 11), practical support(items 9–10), patient responsibilities (item 8), and quality of communication between patients and healthcare providers on healthcare visit (item 12) and family coping (item 13). For the navigation subscale, questions on navigating health and treatment (item 14), navigating communication methods (item 15), navigating the differences between healthcare professionals’ roles (item 16), financial resources (item 17), navigating cancer-related information between providers (item 18), navigating primary care provider appointments (item 19), and navigation wait time between test or treatment (item 20).

#### Primary outcome: depression

The Patient Reported Outcomes Measurement Information Systems (PROMIS) Emotional Distress Short Form v1.0, Depression 4a was used to measure depression [27]. This 4-item scale has demonstrated high reliability and construct validity [28, 29]. It measures respondents’ negative mood, reduced positive affect, negative self-view, and social connectedness over the past week. Each item uses a 5-point Likert response scale ranging from “Never” to “Always.” Raw scores were then converted to T scores, with a T scores of 50 and standard deviation of 10 representing the average score for the general US population. T scores of 55 or above were considered clinically significant depression based on established cutoffs [30]. This instrument has been tested and validated to measure depression in people with various medical illnesses, including but not limited to cancer, cardiac disease, transplant, and orthopedic surgery recipients [31].

#### Demographic and disease-related variables

Demographic variables included gender, marital status, annual household income, health insurance type, and caregiver status. All demographic variables were recoded as binary categories, with a score of “1” representing male gender, married, annual household income equal to or above 60,000 United States dollars (USD), holding commercial health insurance, and having an informal caregiver. A score of “2” represented female, unmarried, with an annual household income of less than 60,000 USD, holding government-sponsored health insurance (including Medicare enrollees), and having no caregiver. Disease-related variables were cancer stage and age at cancer diagnosis.

### Statistical analysis

Exposure variables included 22 items from the CCCQ-P, five demographic items, and two clinical variables, and the outcome measure was the PROMIS depression T score. R programming was used for statistical calculations [32]. Frequency distribution, including numbers, percentages, mean, and standard deviations, were used to illustrate participants’ baseline characteristics. Machine learning models, including the Recursive Partitioning and Regression Trees (RPRT) and Generalized Boosted Regression Models (GBMs), were selected to assess sociodemographic, clinical, and CCC factors and to identify the most important correlates of depression [33]. GBMs is a flexible, nonparametric ensemble method that allows for the inclusion of numerous correlated variables without the same inflation of type I error risk associated with traditional regression models. This approach is particularly helpful for exploring complex, potentially nonlinear relationships. Although the study is correlational, GBMs enabled us to identify the most influential care coordination factors associated with depression for future hypothesis-driven research.

The repeated K-fold cross-validation with five folds, repeated calculation twice, 69% for training, and 31% for testing splits were used as the tuning grids for the Recursive Partitioning and Regression Trees. The hyperparameter was set to have a minimum of five observations in each terminal node and at least 20 observations to allow a subsequent split. After the removal of missing data, 339 observations were retained for analysis. The “bag impute” function from the Caret (Classification and Regression Training) package replaced missing observations in the exposure variables with those in the bagged trees to reduce the loss of observations [34]. For the Generalized Boosted Regression Models, repeated K-fold cross-validation using five folds and repeated calculation twice, 70% for training and 30% for testing sets were used as the tuning grids. The hyperparameter was set to test a range of 100 to 3000 trees, a maximum interaction was set at 2–3 between variables, a model learning rate (lambda) of 0.01, and a minimal observation of five in each terminal node.

### IRB approval

The UCLA Institutional Review Board (IRB) reviewed and approved this study under protocol number #19–000798.

## Results

Of the 1173 potentially eligible HNC survivors invited to participate, 371 responded, yielding a response rate of 31.6%. Of these, 24 individuals were deemed ineligible or decided not to participate. In comparing respondents to non-respondents, no significant differences were observed in time since diagnosis, age at diagnosis, gender, cancer stage, marital status, or alcohol use history (all *p* > 0.05) Significant differences were found in race, Hispanic ethnicity, smoking status, and primary tumor site, with non-White individuals (17.5%, *p* < 0.001), those identifying as Hispanic (16.8%, *p* = 0.002), and current smokers (16.5%, *p* = 0.002) more likely to be non-respondents. Survivors with tonsillar cancer were more likely to participate (37.7%, *p* = 0.02).

A total of 347 head and neck cancer (HNC) survivors completed the survey, all of whom had undergone surgery, radiation, chemotherapy, or a combination thereof. Fewer than 20 participants were still receiving targeted therapy, immunotherapy, or both at the time of the survey. The majority of respondents were male (66.6%), married (70.6%), had an annual household income of $60,000 or more (65.1%), and did not report having a caregiver (69.5%). The mean age at the time of the survey was 65.6 years (SD = 11.3), and the mean age at diagnosis was 61.8 years (SD = 11.7). Participants were, on average, 4 years post-diagnosis (range = 13.87 to 375.55 months). All respondents had either government (46.7%) or commercial (53.3%) health insurance (Table 1).

**Table 1:** Sample characteristics

	**Mean**	**SD**
**Current age (years)**	65.6	11.3
**Age at diagnosis (years)**	61.8	11.7
	***N***	Percentage (**%**)
Less than 55 years	85	25.5
55 to 65 years	119	34.3
Above 65 years	143	41.2
**Gender**	***N***	**%**
Male	231	66.6
Female	116	33.4
**Marital status**	***N***	**%**
Married	245	70.6
Unmarried	101	29.1
**Annual household income**	***N***	**%**
Equal or above $60,000 USD	226	65.1
Below $60,000 USD	80	23.1
**Caregiver**	***N***	**%**
With a caregiver	105	30.3
Without a caregiver	241	69.5
**Staging**	***N***	**%**
0 to II	179	51.6
III to IV	146	42.1
**Health insurance**	***N***	**%**
Government	162	46.7
Commercial	185	53.3
**Race**	***N***	**%**
White	283	81.5
Asian	21	8.5
Hispanic	14	4
Black	6	1.7
Other	19	5.5
**Education**	***N***	**%**
Less than high school	6	1.7
High school graduate	25	7.2
Some college	111	32
College graduate	123	35.4
Postgraduate	80	23.1
**Treatment modality**	***N***	**%**
Surgery	275	80.2
Chemotherapy	164	47.8
Radiation therapy	272	79.3
Targeted therapy	25	8.3
Immunotherapy	37	11.5
**Primary tumor site**	***N***	**%**
Pharynx	120	34.6
Sinus, salivary, other	39	11.2
Tongue, oral cavity	188	54.2
**Time since diagnosis (month)**	**Mean**	**Range**
	44.44	13.87–375.55

The average CCCQ-P total, communication subscale, and navigation subscale scores were 74.6 (*SD* = 14.6), 47 (*SD* = 10.2), and 27.6 (SD = 5.7), respectively. Average scores for overall CCC rating (item 21) and care satisfaction (item 22) were 8.3 (SD = 2) and 8.9 (SD = 1.7), respectively. The detailed scores for the individual CCCQ-P items are displayed in Table 2.

**Table 2:** Cancer Care Coordination Questionnaire for Patients (CCCQ-P) raw scores

Item number	Item name	Mean	SD
Item 1	I knew the warning signs and symptoms I should watch for to monitor my health	3.48	1.13
Item 2	I always knew what tests, treatments and follow up were planned for me	3.85	1.04
Item 3	I knew whether chemotherapy or radiotherapy were suitable for me	3.74	1.00
Item 4	I always knew the reason why I was having a test or treatment	4.18	0.74
Item 5	I was fully informed about the advantages and disadvantages of any additional treatments that were relevant to me	3.87	1.02
Item 6	I had access to all the additional services (e.g., stoma therapy, counselling, cancer support groups, nutritional advice) that I needed	3.83	1.03
Item 7	I had sufficient help from staff with dealing with the emotional impact of my cancer	3.62	1.06
Item 8	I had a good understanding of the things I was responsible for to help my treatment plan run smoothly	4.07	0.82
Item 9	I had sufficient help from staff with practical arrangements	3.98	0.86
Item 10	I was fully informed by staff about my financial entitlements (e.g., Medicare and health fund claims, travel allowances etc.)	3.45	1.12
Item 11	The health professionals looking after me always picked up on whether I was feeling anxious or down	3.60	1.03
Item 12	How often were you asked how your visits with other health professionals were going?	3.17	1.14
Item 13	How often were you asked how well you and your family were coping?	2.94	1.21
Item 14	How often were you unsure who you should contact if you had concerns about your health or treatment plan?	3.78	1.04
Item 15	How often were you unsure who to call out of business hours if you had a problem?	3.88	1.09
Item 16	How often were you confused about the roles of the different health professionals involved in your care?	4.01	1.00
Item 17	How often was it difficult to meet the financial costs associated with your health care?	4.13	1.08
Item 18	How often did you feel that health professionals looking after you were not fully informed about your history and progress?	4.03	1.00
Item 19	How often did you have difficulty getting an appointment with your GP?	4.32	0.84
Item 20	How often did you have to wait too long to get the first available appointment for a test or treatment?	4.06	0.87
Item 21	In general, how would you rate the co-ordination of your care?	8.36	1.99
Item 22	Overall, how would you rate the care you have received?	8.92	1.69

Items 1–20 use a 5-point Likert response scale ranging from 1 to 5, items 21–22 ranges from 1 to 10. Higher scores indicate higher satisfaction (items 14–20 were reverse coded)

The average depression score for the respondents was 48.9, with a standard deviation of 8.6. A majority of respondents (74.6%, *n* = 253) had PROMIS depression T scores below the threshold of depression (T score < 55), while 25.4% (*n* = 86) met the cut-off score (T score = or > 55). The missing data in each variable was below 5% except for the overall CCC rating (item 21; 5.8%), care satisfaction rating (item 22; 5.5%), annual household income (11.5%), and cancer stage (6.3%).

The recursive partitioning tree in Supplemental Material 2 illustrates the process of splitting and identifying the most important exposure variables. The training and testing datasets achieved an accuracy and area under the curve (AUC) of 0.85/0.87 and 0.73/0.64, respectively, using the recursive partitioning tree. The Generalized Boosted Regression Models produced the best accuracy of 0.78 with 300 trees and a 3-way interaction. The accuracy and AUC for the training and testing models using stochastic gradient boosting were 0.84/0.92 and 0.76/0.74, respectively.

The results from the Generalized Boosted Regression Models indicated that age at cancer diagnosis (importance score = 100) and confusion about the roles of health professionals involved in their care process (item 16, importance score = 99.1) were identified as the most significant correlates of depression. The overall rating of care coordination (importance score = 62.2) and caregiver status (importance score = 47.8) were moderate-strong correlates, while practical support from staff (importance score = 40.9), annual household income (importance score = 40.7), and information about treatment and financial entitlements (importance score = 38.4 and 38.3, respectively) were moderately important correlates. Other variables displayed lower but meaningful importance, such as cancer staging (importance score = 36.9) and overall healthcare rating (importance score = 31.3) (Table 3).

**Table 3:** The ranking of factors associated with depression in head and neck cancer survivors

Importance	Variable	Score
1	Age at diagnosis	100
2	Item 16: How often were you confused about the roles of the different health professionals involved in your care?	99.12
3	Item 21: In general, how would you rate the coordination of your care?	62.19
4	Caregiver	47.81
5	Item 9: I had sufficient help from staff with practical arrangements	40.94
6	Annual household income	40.69
7	Item 5: I was fully informed about the advantages and disadvantages of any additional treatments (e.g., radiotherapy, chemotherapy, or hormonal therapy) that were relevant to me	38.45
8	Item 10: I was fully informed by staff about my financial entitlements (e.g., Medicare and health fund claims, travel allowances, etc.)	38.33
9	Staging	36.91
10	Item 22: Overall, how would you rate the care you have received?	31.3

Generalized Boosted Regression Models (GBMs)–stochastic gradient boosting

Score: higher scores suggest that the variable is more important in explaining depression

**Fig. 1:**
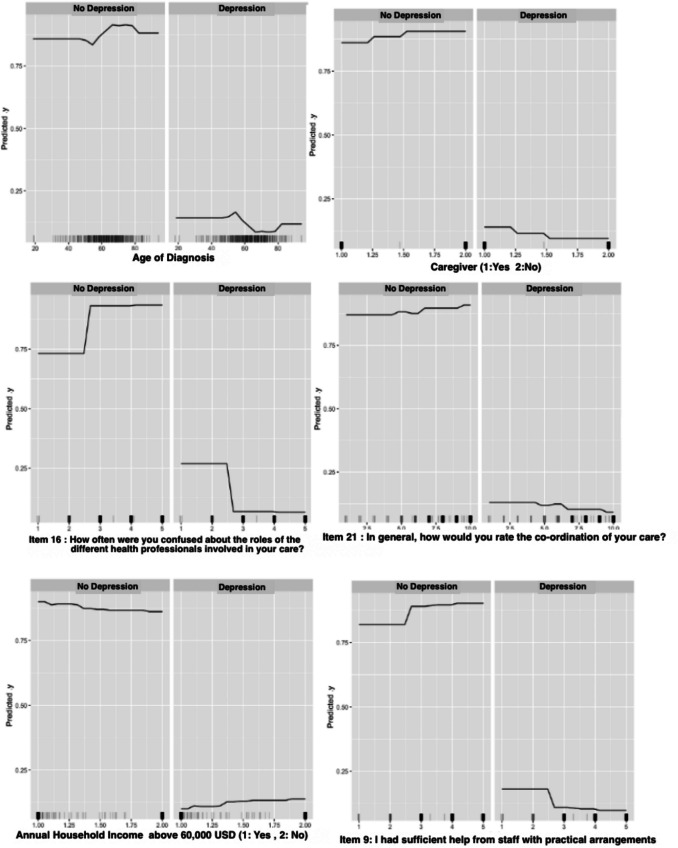
Partial dependence plots (PDPs) to understand the direction of the relationship between exposures and outcome. The PDPs revealed non-linear, negative relationships between participants’ degree of clarity of the healthcare professionals’ role involved in their care (item 16), sufficient help from staff with the practical arrangement (item 9), the overall cancer care coordination (CCC) rating (item 21), and depression. Caregiver and the annual household income are variables with binary categories (1: caregiver presence and annual household income equal to or above 60,000 USD; 2: no caregiver and annual household income below 60,000 USD). Lower-income and caregiver presence was associated with a higher likelihood of depression

Partial dependence plots were used to examine the shape and direction of the relationships between the most important correlates and depression, revealing that age at cancer diagnosis, confusion about healthcare professionals’ roles during their cancer care journey (item 16), overall CCC rating (item 21), and support from care staff with practical arrangements (item 9) had negative non-linear relationships with depression. At the same time, participants who did not have a caregiver and an annual household income equal to or less than 60,000 USD were more likely to be depressed compared to participants without a caregiver and with an annual household income of above 60,000 USD (Fig. 1).

Among participants who met the criteria for depression, there was a linear reduction in depression risk between the ages of 65 and 77. In contrast, being diagnosed with cancer before the age of 55 was associated with higher depression risk. Furthermore, HNC survivors’ clear understanding of the healthcare professionals’ role, a higher overall CCC rating, and support from care staff in meeting their practical needs was associated with lower depression risk among participants who met the depression score cut-off (T score 55) (Fig. 1).

We examined whether baseline characteristics differed between HNC survivors with above- versus below-average scores on item 21, which assessed overall perceptions of cancer care coordination. Survivors with commercial insurance were significantly more likely to report a better experience with care coordination compared to those with government insurance (*p* = 0.04). No statistically significant differences were observed by gender, marital status, income, cancer stage, or age at diagnosis.

## Discussion

This study identified care coordination factors associated with depression among HNC survivors and established a sequence of importance among care coordination, demographic, and clinical determinants of depression during extended survivorship. In this sample of HNC survivors who were on average 4.4 years post-diagnosis, 25% exceeded the clinical threshold for depression. Findings indicated that being diagnosed with cancer at middle adulthood were the most important correlates of depression. These findings suggest that universal depression screening and interventions to help patients understand their complex care teams may be beneficial to study to improve the mental well-being of HNC survivors.

Middle-aged HNC survivors demonstrated higher depression risk than older adult survivors, contrasting with existing literature that has primarily focused on mental health risks in younger and older adult populations [35–38]. This vulnerability may reflect the unique challenges faced by middle-aged adults who are simultaneously managing peak career responsibilities, family caregiving obligations, and their own complex medical needs following HNC treatment. The potential functional impacts of HNC, including changes in speech, swallowing, and facial appearance, may also be particularly challenging for individuals in middle age, potentially affecting employment and earning capacity, social relationships, and family roles [39].

Beyond demographic factors, our study found confusion about healthcare team member roles emerged as the second most important correlate of depression, highlighting the critical importance of clear communication and role delineation in cancer care. This finding is particularly significant given that HNC care involves uniquely complex, multimodal treatments requiring coordination among surgical oncologists, radiation oncologists, medical oncologists, and multiple rehabilitation specialists including speech-language pathologists, nutritionists, and social workers [40]. The absence of a designated point person or liaison to coordinate care among these providers can substantially increase patient distress and confusion, especially when complications or concerns arise during survivorship when regular contact with the care team diminishes. This finding suggests that survivors’ understanding of their care team structure and knowing whom to contact for specific issues directly impacts their psychological well-being, even during extended survivorship. Our findings also demonstrate the impact of timely access to practical support services, including transportation, housing, and financial assistance, on mental health outcomes among HNC survivors.

With an average CCCQ-P total score of 74.6 (SD = 14.6), participants indicated poorer perceptions of care coordination compared to previous reports in the literature among lung cancer patients assessed within 2 to 12 months post-diagnosis (mean total score 78.1) [26] and gastrointestinal cancer survivors evaluated an average of 3.3 months post-treatment (mean total score 76.6 ± 11) [41]. Notably, survivors with government health insurance in our study demonstrated particularly elevated levels of unmet CCC needs, reflecting the intersection between insurance coverage and care coordination quality in the US healthcare system. Unlike the intensive coordination typically needed during diagnosis and acute treatment, HNC survivorship care frequently involves fewer healthcare touchpoints and disconnected specialists, creating opportunities for care coordination to deteriorate. According to our findings, ensuring that HNC survivors understand the members of the care team and their roles is a critical aspect of care coordination for supporting survivors’ mental health. HNC survivors may be unsure of where or from whom on the team to seek support. Given the ongoing rehabilitative and psychosocial issues many HNC survivors experience, proactive care coordination strategies are needed during the extended survivorship phase to prevent care gaps.

These results support the use of care coordination interventions in survivorship care, such as patient navigation and comprehensive case management models [42], which have been shown to enhance care continuity, improve quality of life, and reduce high-cost healthcare utilization [43, 44]. Although these interventions vary in format, they share a common focus on identifying individual patient needs and maintaining ongoing patient engagement, recognizing that patient circumstances evolve throughout the care trajectory. Patient navigators or case managers can serve as consistent points of contact who clarify healthcare team roles, promote communication between providers, and ensure survivors understand how to seek support and access resources. To facilitate targeted interventions and timely referrals, navigation and case management care models can also incorporate standardized screening tools, such as the NCCN distress thermometer and problem list, to identify specific areas of concern across physical, practical, psychological, and spiritual domains [45, 46]. For HNC-specific assessment, the patient concerns inventory (PCI), which includes dental health/teeth and swallowing/saliva issues among others, represents a targeted option [47, 48]. Care coordination interventions can also help avoid information asymmetry, or discrepancies of understanding between healthcare providers and patients, and bridge communication gaps through structured assessments, regular check-ins, and patient education [49].

To our knowledge, this is the first study to examine patient-reported care coordination experiences and their relationship to depression among HNC survivors. Although the CCCQ-P evaluates patients’ perceived care coordination rather than their actual care processes, these perceptions play a vital role in shaping patient satisfaction, engagement, and healthcare trust. Discrepancies between what patients perceive and what is documented in their clinical records may signal issues with communication, the complexity of their care, or unmet information needs. Future studies could investigate how perceived care coordination impacts additional survivorship outcomes, such as patient satisfaction, quality of life, return to work, role satisfaction, or post-traumatic growth. Furthermore, while this study focused on care coordination needs, we acknowledge that psychological, physical, and social needs also affect mental health and should be considered in future models that address survivorship well-being from multiple angles.

### Study strengths and limitations

Applying machine learning methods offers a robust alternative to traditional statistical approaches, particularly in addressing key computational limitations such as inflated standard errors when modeling numerous variables in studies with modest sample sizes, and violations of assumptions stemming from data nonlinearity and skewed distributions. GBM builds an ensemble of shallow decision trees sequentially, with each tree trained to reduce the error left by the previous one by minimizing a specific loss function. Although individual splits are guided by impurity measures, the overall model focuses on gradually reducing errors, which helps identify variables with the most impact. To deal with class imbalance in variables with few categories, a down-sampling technique was used to boost the model’s robustness and generalizability. By leveraging these methods, we were able to detect specific CCC barriers faced by HNC survivors, facilitating the development of precision interventions aimed at reducing depression risk through improved care coordination.

This study has several limitations. First, its single-site, cross-sectional design and reliance on self-reported surveys introduce potential recall and selection biases and hinder the ability to make causal inferences. Future studies should employ longitudinal or quasi-experimental designs with careful adjustment for confounders to confirm the directionality and mechanisms of these associations. Second, the CCCQ-P lacks a fixed recall window, and participants may have reflected on different phases of their cancer care, adding variability in responses. Third, the sample primarily included older, insured, English-speaking White participants, limiting generalizability and excluding insights from survivors with limited English proficiency or without insurance. Moreover, the absence of randomized control trial data on utilizing distress thermometer and problem list and without established mental health referral plannings limits our ability to link perceived care coordination with actual service use. Lastly, the COVID-19 pandemic may have contributed to the lower response rate in our study. However, a sub-analysis examining nonresponse bias revealed no significant differences in most clinical and demographic characteristics, except that racial minority participants and current smokers were less likely to respond to the survey.

### Clinical implications

Findings suggest a need for care coordination strategies beyond acute HNC treatment, especially to ensure that survivors understand their care teams. HNC survivors continue to face complex follow-up care, persistent side effects, and challenges in accessing supportive services—issues that can significantly impact their mental health and may be improved through better care coordination. The study findings point to a need to develop and evaluate survivorship care models that incorporate care coordination strategies to reduce health care-associated distress and long-term psychological burden observed in this population. Such models should include clear pathways for addressing identified concerns and accessing supports and resources with a specific point person, when possible, and detailed information about the structure and roles involved in survivorship care.

## Conclusion

This study demonstrates that specific aspects of cancer care coordination significantly influence psychological well-being among HNC survivors during the extended survivorship phase. The identification of younger age at diagnosis and poor healthcare team role clarity as primary depression correlates provides actionable targets for intervention development. As the number of cancer survivors is projected to reach 26 million by 2040 [50], care coordination strategies need to be prioritized in survivorship care models to bridge oncology, primary care and specialty teams, support the complex, evolving needs of survivors and optimizing their long-term health outcomes.

## Supplementary Information

Below is the link to the electronic supplementary material.

Supplementary file1 (DOCX 291 KB)

## Data Availability

The datasets generated during the current study are available from the corresponding author on reasonable request.
