# Supplementary material for: Cancer care coordination determinants of depression in head and neck cancer survivors
Source: Support Care Cancer. 2025 Aug 20;33(9):798. doi: 10.1007/s00520-025-09847-2 (PMC12364750; doi:10.1007/s00520-025-09847-2)
Supplement: Supplementary file 1 — Supplementary file1 (DOCX 291 KB) [file 520_2025_9847_MOESM1_ESM.docx]

**Supplemental Materials**

Supplemental Material 1. Theoretical Framework on Factors Influencing Health Outcomes (Adapted from Gelberg and Anderson’s Behavioral Model for Vulnerable Population)

**Supplemental Material 1 Legend. An individual's health outcome is driven by predisposing characteristics, enabling resources, and their perceived or evaluated care needs. Applying the framework to this study, age of cancer diagnosis, gender, and cancer stage are predisposing characteristics. Enabling resources include marital status, annual household income, and insurance type. Patients’ perception of cancer care coordination represents their perceived needs for care.**

Supplemental Material 2. Recursive Partitioning and Regression Trees: Splitting Process and Variable

of Importance
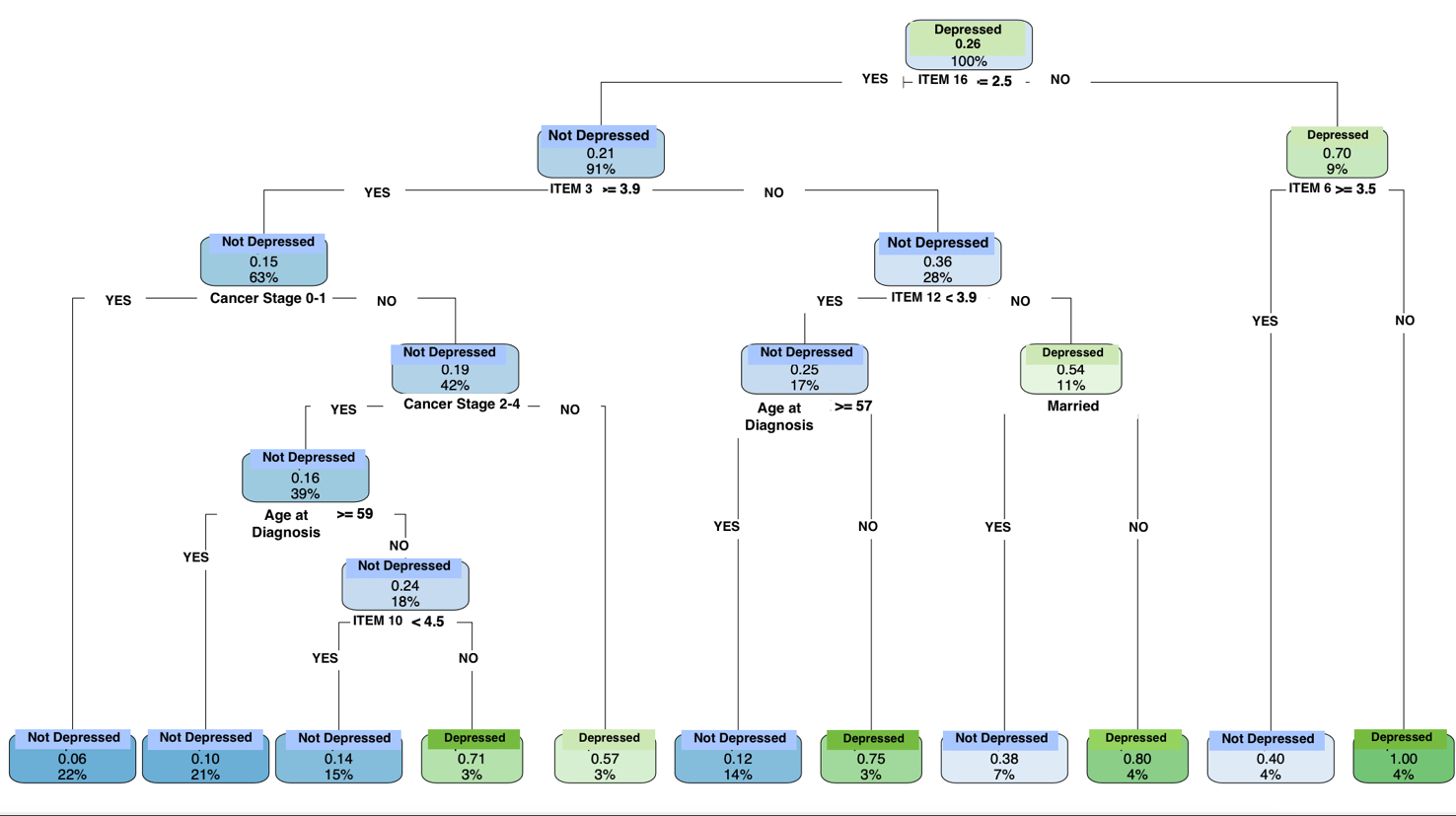


Supplemental Material 2 Legend. The first and second splits occurred at item 16, item 3, and item 6, revealing their importance in predicting depression.

Item 3: I knew whether chemotherapy or radiotherapy was suitable for me

Item 6: I had access to all the additional services (e.g., stoma therapy, counseling, cancer support groups, nutritional advice) that I needed

Item 10: I was fully informed by staff about my financial entitlements (e.g., Medicare and health fund claims, travel allowances)

Item 12: How often were you asked how your visits with other health professionals were going?

Item 16: How often were you confused about the roles of the different health professionals involved in your care?
